# Predictors of Prolonged Hospitalization in Patients with Fever

**DOI:** 10.7759/cureus.789

**Published:** 2016-09-18

**Authors:** Shoab Saadat

**Affiliations:** 1 MBBS, Resident Nephrology, Shifa International Hospital, Islamabad, Pakistan

**Keywords:** predictors of prolonged hospitalization, hospitalization, platelet count, length of stay, fever, total leukocyte count, undiagnosed fever, reduced length of stay (los), stay, febrile fever

## Abstract

Objective: The study was conducted to analyze the predictors of prolonged hospitalization in patients with fever.

Patients and Methods: This was a prospective cohort study conducted from July - December 2015 at Ayub Teaching Hospital, Pakistan. Convenience sampling was used to enroll the patients who visited the hospital during the study duration. A sample size of 115 patients was calculated. It included patients who presented with a new onset fever which started in the last month, and the cause of fever was undiagnosed at the time of admission. Critical patients were excluded. Data for more than 30 variables was collected on a pro forma. Univariate regression methods were used to analyze the data in the Statistical Package for Social Sciences (SPSS), version 23.

Results: A total of 115 patients were analyzed. Males constituted 66/115 (57.4%). The mean age for patients was 43.6 years (standard deviation (SD) = 20.2). On admission, low platelet counts (p = 0.001), high erythrocyte sedimentation rate (ESR) counts (p = 0.007), a high total leukocyte count (TLC) (p = 0.029), and involvement of nervous (p = 0.021), cardiovascular (p = 0.04), respiratory (p = 0.043), gastroenterological (p = 0.042), hematological (p = 0.028), or urogenital system (p = 0.016) were associated with a longer stay in the hospital.

Conclusion: Patients with an undiagnosed and new onset fever will have a longer hospital stay if, on admission, they have low platelet counts, a higher ESR, a high TLC, or involvement of nervous, cardiovascular, respiratory, gastrointestinal, hematological, or urogenital systems. An early identification of risk factors can lead to better treatment and may also lead to a decreased hospital stay.

## Introduction

A large number of patients are admitted all over the world who present with an undiagnosed and new onset fever, most of them are found to have an infectious etiology, followed by autoimmune causes [1]. Most of these patients have an infective cause and they belong to third world countries where there is always a pressure of finances on them. Longer admission times for the patients admitted with fever have been shown to be causing a substantial financial burden on a country’s economy, too [2]. There is also a huge social and psychological impact on the patients themselves and their families, along with an increased hospital stay. Depression is a common problem, which is found to be associated with patients having prolonged hospitalizations [3]. All this is coupled with the fact that prolonged hospitalizations are associated with a greater incidence of hospital-acquired infections, which in turn can lead to an even longer hospital stay [4]. This assumption is true for both the infective and non-infective causes of undiagnosed fever [5].

Since lesser work has been done on the identification of risk factors associated with prolonged hospital stays in Pakistani patients with undiagnosed fever, there was a need for such a study. This study may help in the generation of bedside tools or scoring systems that will risk stratify the patients based on the presence or absence of risk factors. There have been many studies published internationally that have mentioned elevated admission total leukocyte counts (TLC) as a valuable predictor of mortality and a tool for risk stratification during hospitalization [6].

A longer hospitalization is also associated with more inpatient complications [7]. Therefore, the present study was conducted to identify early risk factors associated with a prolonged hospital stay in patients with undiagnosed fever. 

## Materials and methods

This was a prospective cohort study conducted from July to December 2015 at Ayub Teaching Hospital (ATH), a large public sector hospital of Pakistan. The study was approved by the hospital administration and community medicine department. There was no IRB office in Ayub Teaching Hospital at the time of commencement of this study. It included the patients who visited the hospital during the six months of the study period and fulfilled the inclusion criteria. All the patients with a history of new onset fever measured to be greater than 98° Fahrenheit that had started within the previous month and who had remained undiagnosed at the time of admission, had not been to any other facility, and were a candidate for admission were analyzed. All the patients included in the study gave their oral consent for the data collection. Those patients who were critical or having septic shock that were candidates for medical or surgical ICU, who had used antibiotics in the past for this episode of fever, or who did not consent were excluded from the study. Convenience sampling was used to select the patients who visited the hospital during the study duration. A sample size of 115 patients was calculated, taking the proportion of undiagnosed fever in the South Asian population to be 5% and the estimation error to be 0.04 using World Health Organization (WHO) calculator [8].

A study proforma was used to collect all the data and was administered by a health care provider. The study variables included demographic data, including age, sex, date of admission, source of admission, socioeconomic status, and district. Several predictor variables were recorded, such as the presence of rigors and chills, body aches, values of total leukocyte count, platelet count, erythrocyte sedimentation rate (ESR), temperature, blood pressure (BP), pulse rate, hemoglobin (Hb) level, presence of diabetes mellitus, personal exposure to tuberculosis (TB) infection in past, hepatomegaly or splenomegaly found on clinical examination, jaundice, white blood cells in routine urine examination, etc. System involvement referred to the presence of meningitis or fits for the central nervous system (CNS); myocardial infarction or arrhythmias for the cardiovascular system (CVS); pneumonia for respiratory system; diarrhea and hepatitis for the gastrointestinal tract (GIT); leukemia and lymphomas for hematological system; and urinary tract infections and pelvic inflammatory disease for the urogenital system. These predictor variables were recorded at the time of admission or within the first three days after admission.

The analysis was performed using the Statistical Package for Social Sciences (SPSS), version 23 (IBM SPSS Statistics, Armonk, NY). A total of 30 variables were collected and assessed. Descriptive analysis was done using means for continuous and percentages for categorical variables. Predictive modeling techniques were used for making inferences using an alpha value of 0.05. Multiple regression was used with duration of hospital stay as a response variable while the predictors mentioned above were put in the model as independent variables. The accuracy of the model was assessed using Akaike Information Criterion Corrected (AICC). Residuals obtained from the final model were negative for heteroscedasticity.

## Results

A total of 115 patients were analyzed. In this study, there were 66 males (57.4%) in the total population while the rest were females. The mean age of the patients was 43.6 (SD = 20.2) years. A total of 82 out of 115 (71.3%) patients had no education at all; 84 out of 115 (73%) belonged to a lower class with an earning of less than Rs. 20,000/month by the whole family. All the patients presented with fever while 73% of them had additional complaints of rigors and chills. Fourteen of the 115 patients (12.2%) had an exposure to tuberculosis infection. Thirty-two out of 115 patients (27.8%) either had a hepatomegaly or a splenomegaly at the time of presentation. A high ESR count that was greater than 20 was seen only in nine (8%) of the patients. After the admission, 11 (9.6%) patients tested positive for a malaria parasite. No one tested positive for Brucella antibodies or for a polymerase chain reaction (PCR) test for the dengue fever. The mean value for the hemoglobin variable was 11.33 g/dl (SD = 1.8). The mean hospital stay was 5.7 days (SD = 2.6).

Continuous variables like age, hemoglobin, duration of admission, and platelet counts were normally distributed while a few patients showed very high TLC counts and were marked as outliers but were included in the study as their exclusion had little effect on the study results.

**Figure 1:**
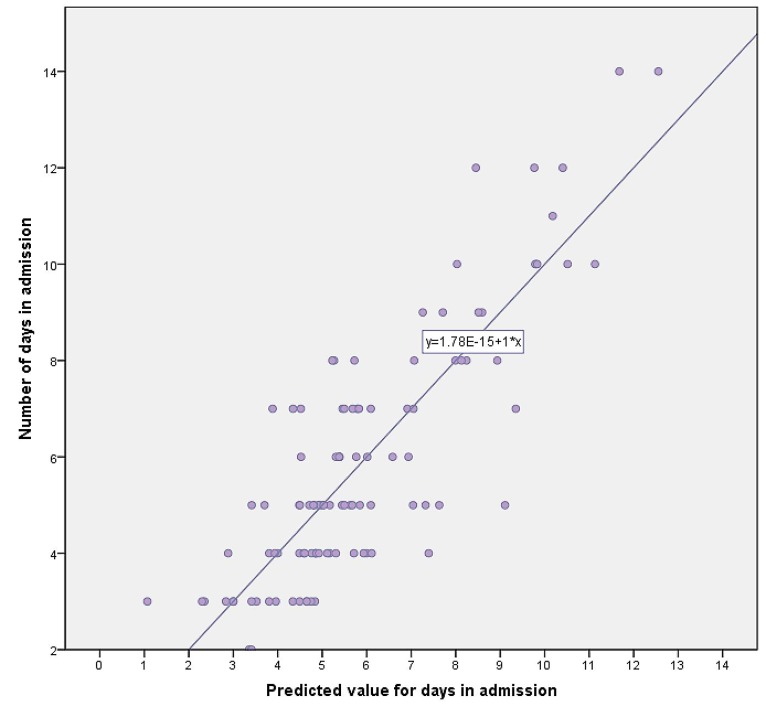
Predicted duration of hospital stay compared to the actual duration of hospital stay The figure summarizes the relationship between the predicted values of how many days a patient is expected to remain hospitalized based on the regression model on x-axis versus how many days that patient actually remained hospitalized. It shows that observations follow a linear pattern suggesting a close correlation, thus, a high predictive ability of the regression model. In other words, it shows that all the variables used in building this model are strong predictors of hospital stay.

The inferential and predictive part of the analysis included building a multiple regression model (Figure 1) in which the hospital stay duration was selected as the response variable. Hospital stay in days was taken to be from the day the patient was admitted for the treatment and workup of fever to the day the patient was discharged in a clinically and vitally stable condition, with or without any definitive diagnosis. 

Age, sex, socioeconomic status, the presence of rigors and chills, body aches, ESR count, the presence of any other systemic complaint, BP, pulse, temperature, TLC count, and platelet counts were chosen as predictor variables and were introduced in the model one by one and in different combinations. Other suspected confounders or disease conditions, like diabetes mellitus, hypertension, or underlying nutritional deficiency disorders, were also adjusted for in the model to exclude any confounding effect on the main predictor variables. This was then repeated for the diagnosed causes of fever one by one. It was done to obtain the most stable and accurate model with the least possible variables while keeping in mind the compromise in the degree of freedom. This model was generated using the entry method. The final results (Table 1) show an R-square value of 0.716; thus, the model was able to explain 71.6% variance in the response variable.

**Table 1:** Tests of Between-Subjects Effects Note: All the predictors with a level of significance < 0.05 have been shown above. Other predictors not shown here were also included in the derivation of the final model. Dependent Variable: Duration of stay in hospital Abbreviations: df = degree of freedom; Sig = significance level a: R Squared = .716 (Adjusted R Squared = .533)

	Type III Sum of Squares	df	Mean Square	F	Sig.	
Corrected Model	510.983^a^	41	12.463	3.926	.000	
Intercept	5.503	1	5.503	1.734	.193	
Platelet Count	36.041	1	36.041	11.353	.001	
ESR Count	34.106	2	17.053	5.372	.007	
Education Level	29.452	3	9.817	3.093	.033	
Total Leukocyte Count	15.848	1	15.848	4.992	.029	
Corrected Total	714.151	105				
Error	203.168	64	3.175			
Total	4364.000	106				

**Figure 2:**
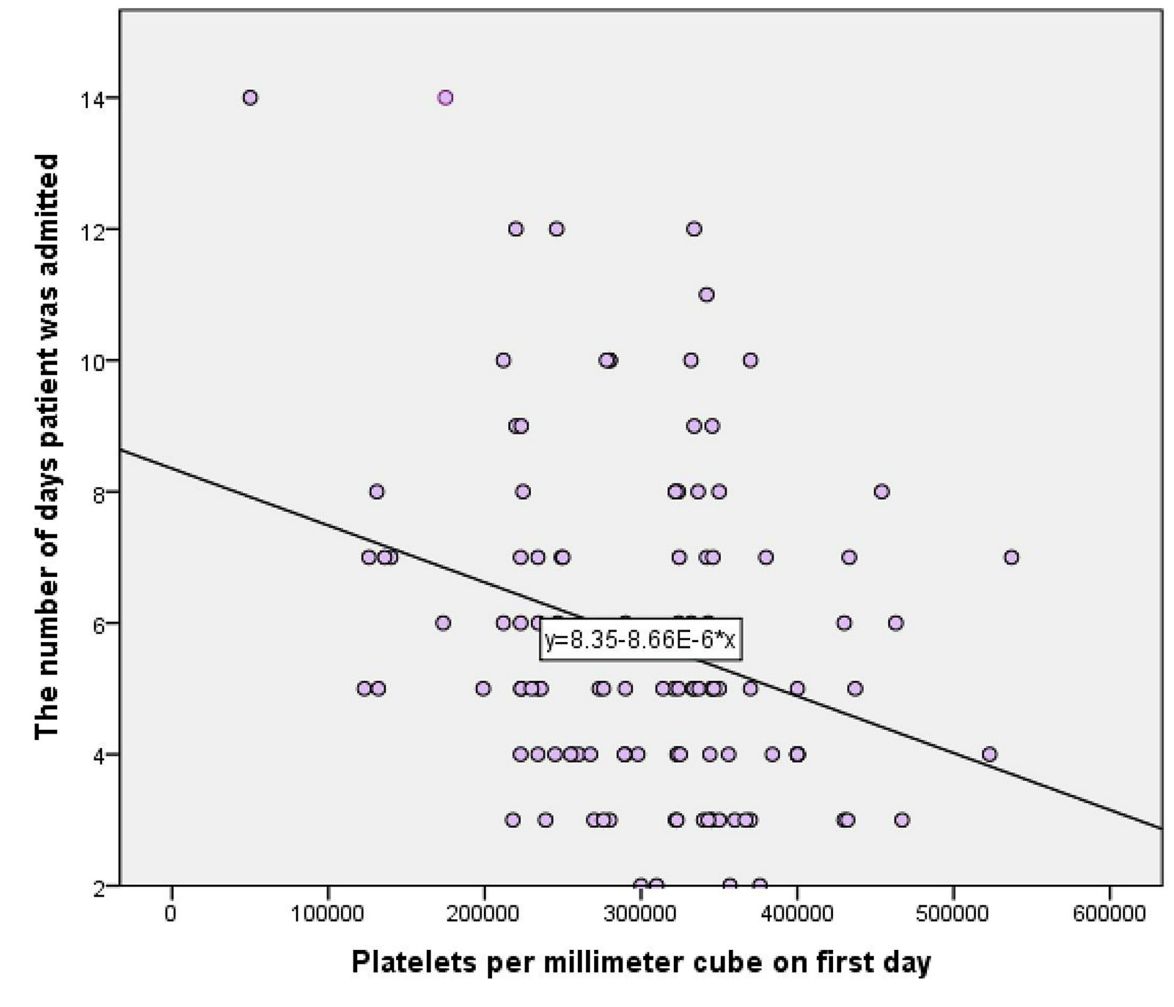
Estimated means chart showing the relationship of platelet count to the duration of hospital stay This chart summarizes how a decrease in platelet count is associated with increased hospital stay in days.

Platelet count was the most important predictor variable that contributed to the model (p = 0.001), followed by the education status (p = 0.033), ESR count (p = 0.007), and TLC counts (p = 0.029). Socioeconomic status, pulse rate, and history of diabetes mellitus also contributed to the model but insignificantly. A fall in platelet count was associated with a longer duration of stay (p = 0.01) in the hospital as was a higher ESR count (p = 0.007) (Figure 2). 

The presence of nervous (CNS) (p = 0.021), cardiovascular (CVS) (p = 0.040), respiratory (p = 0.043), gastroenterological (GIT) (p = 0.042), hematological (p = 0.028), or urogenital (p = 0.016) system involvement was also significantly associated with a longer duration of hospital stay (Table 2). A high TLC count, greater than 10,000 per microliter (p = 0.029) on admission, was also associated with a longer duration of stay in the hospital. Model residuals were tested for normality and a P-P plot was obtained. There were no differences between age or sex. 

**Table 2:** Parameter Estimates Dependent Variable: Length of hospital stay Abbreviations: B = beta coefficient; Sig = significance level a: Computed using alpha = .05

	B	Std. Error	Sig.	95% Confidence Interval		Observed Power^a^
Intercept	28.493	22.064	.201	-15.585	72.572	.246
Urogenital involvement	4.368	1.763	.016	.845	7.890	.684
Nervous system involvement	3.786	1.595	.021	.600	6.972	.647
Hematological involvement	3.814	1.694	.028	.429	7.199	.601
Cardiovascular involvement	3.766	1.793	.040	.185	7.347	.544
Gastrointestinal involvement	3.568	1.723	.042	.127	7.010	.532
Respiratory involvement	3.061	1.483	.043	.099	6.022	.529

## Discussion

This study, which was of a prospective cohort design, included a total of 115 patients. Some highlights of the findings included the role of multiple predictors in determining how long a patient would stay in the hospital after they present with the complaints of undiagnosed and new onset fever. Among several predictors, the most significant ones included platelet count, ESR count, and the involvement of the nervous, cardiovascular, respiratory, gastrointestinal, hematological, or urogenital system.

A higher TLC count was also related to a greater duration of stay in the hospital. Predictors of education level, socioeconomic status, and a history of diabetes mellitus contributed to a lesser extent with insignificant p values.  

In a 2016 study conducted at a Korean hospital, patients who had community-acquired pneumonia as a cause of fever were assessed. Prolonged hospitalization was found to be associated with lower Hb levels and a higher blood neutrophil fraction on admission [9]. A similar finding for low Hb was seen in another study as well [10].

Our study showed no significant relationship between Hb and duration of hospitalization. This may be due to a general trend of lower Hb values (< 11 g/dl) among the Pakistani population [11]. An increased white cell count was seen to be correlated with the duration of hospital stay as was the case in a study done by Naess, et al. in 2014 [12] and in another one done in 2011 by Azab, et al. [13]. In an earlier Pakistani study, neutropenia in patients with dengue fever prolonged hospitalization [14]. Since our study included a wide spectrum of causes of fever, a general trend of rising TLC was found to be associated with a prolonged hospital stay. In a more recent study by Williams, et al., C-reactive protein (CRP), but not the TLC, was found to be associated with an increased hospital stay in patients with fever [15]. In another study, patients with prolonged fever were assessed. It showed older population to be associated with a longer hospital stay [16]. This was in contrast to our study where age had no significant association. Malaria was found to be a common cause of fever and was associated with increased hospital stays irrespective of the parasite type. This is in agreement with an earlier study [17].

In our study, thrombocytopenia was significantly associated with a longer hospital stay but this was not the case in some earlier studies [10, 18]. This is possibly because both of these studies looked only at specific causes of fever that can be related to lower platelet counts in advanced stages. Respiratory conditions like pneumonia are among the top conditions associated with prolonged hospitalization [19-20], which was also found in our study.

This study also had its limitations. Although a prospective cohort design meant a less recall and selection bias, a low sample size meant that inferences have to be carefully generalized. Convenience sampling was performed, even though statistical methods were used to adjust for potential confounders. Since all of the patients were studied during the admission period, none was lost to follow-up.

Other potential confounders that could have also been considered include the presence of coagulopathy [19], hematemesis, hematuria, melena, raised alanine transaminase (ALT) [21], serum albumin, CRP level, earlier use of antibiotics, earlier hospitalization, history of any invasive procedure, depression, and confusion. A multivariate model can then be used to better predict the days of hospitalization, and a scoring system can be devised to stratify patients with undiagnosed fever into different risk groups.

Findings from this study can be used to generate a risk stratification tool to be used at the bedside. Such a tool can be used in future studies to enroll patients in larger trials on the basis of risk stratification and compare these different groups with each other. Future studies can validate the findings of this study by including more predictor variables and a larger sample size.

## Conclusions

Patients who present with a new onset undiagnosed fever will have a longer hospital stay if they have low admission platelet counts, a higher ESR, a high TLC, or involvement of nervous, cardiovascular, respiratory, gastrointestinal, hematological, or urogenital systems. An early identification of deranged risk factors can lead to better treatment and may lead to decreased hospital stays.
